# Clinical characteristics and viral load dynamics of COVID-19 in a mildly or moderately symptomatic outpatient sample

**DOI:** 10.1371/journal.pone.0258970

**Published:** 2021-10-21

**Authors:** Amanda Caplan, Kelly W. Bates, Carla Brioni, Aileen Santos, Linda M. Sabatini, Karen L. Kaul, Mercedes R. Carnethon, Janardan D. Khandekar, Philip Greenland

**Affiliations:** 1 Department of Immediate Care, NorthShore University Health System, Evanston, Illinois, United States of America; 2 Department of Pathology and Laboratory Medicine, NorthShore University HealthSystem, Evanston, Illinois, United States of America; 3 Department of Preventive Medicine, Northwestern University Feinberg School of Medicine, Chicago, Illinois, United States of America; 4 Department of Medicine, Center for Personalized Medicine, NorthShore University HealthSystem, Evanston, Illinois, United States of America; CEA, FRANCE

## Abstract

**Background:**

Studies of outpatients with mild or moderate COVID-19 are uncommon. We studied: 1) association of symptoms with reverse transcriptase polymerase chain reaction (RT-PCR) test results; and 2) association of initial RT-PCR cycle threshold (Ct) in relation to duration of RT-PCR positivity in outpatients with mild or moderate COVID-19.

**Methods:**

This was a cohort study of outpatients with confirmed COVID-19 and at least one symptom. Participants had repeat nasopharyngeal swabs and symptom checklists every 3–5 days until two consecutive RT-PCR tests were negative. RT-PCR tests were used to assess viral load. Antibody tests for COVID-19 were performed at 2 weeks, 4 weeks, and 8 weeks after symptom onset.

**Results:**

Twenty-five patients (nine females) were enrolled, ranging in age from 19–58 (median age 28 years). All patients reported at least one symptom, with a median of six symptoms per patient. Symptoms persisted for 6–67 days (median duration 18 days). In all 25 patients, blood samples collected a median of 13 days after symptom onset were positive for SARS-CoV-2 antibodies in 15 (60%). After a median of 28 days following symptom onset, 23/23 patients with available samples tested positive for antibodies. The longest duration of positive RT-PCR test was 49 days from first positive PCR test (Mean = 27.4, SD = 12.5, Median = 24). Initial Ct was significantly associated with longer duration (β = -1.3, SE = 0.3, p<0.01 per 1 cycle higher) of RT-PCR positivity.

**Conclusions:**

In mildly or moderately ill COVID-19 outpatients, RT-PCT tests remained positive for as long as 49 days and test positivity and symptom duration correlated with initial viral load.

## Introduction

COVID-19, caused by SARS-CoV-2, has resulted in severe illness and hospitalization in a relatively small proportion of patients. Most patients remain asymptomatic or develop symptoms that do not require hospitalization. However, studies of outpatients with mild or moderate COVID-19 are uncommon. There is limited information in the published literature about the time period of resolution of symptoms in outpatients and the association of symptoms with viral load, as estimated by the initial reverse transcription polymerase chain reaction (RT-PCR) test results.

A meta-analysis of 77 studies on viral shedding in hospitalized SARS-CoV-2 patients summarized that RT-PCR tests in this relatively ill group of patients remained positive for up to 63 days [1]. To our knowledge, no previous studies of patients whose symptoms remained mild or moderate have been reported that followed patients with multiple repeated oropharyngeal or nasopharyngeal samples and attempted to determine the duration of virus viability after onset of symptoms.

To address gaps related to viral load and association of symptoms with viral load in outpatients with mild-to-moderate disease, we conducted a cohort study with the following aims: 1) to determine the association of symptoms with RT-PCR test results; 2) to study the association of initial PCR cycle threshold (Ct) in relation to duration of RT-PCR positivity in outpatients with mild-to-moderate COVID-19 who did not require hospital admission.

## Methods

The study was IRB-approved by NorthShore University HealthSystem (NSUHS), Evanston, Illinois. Since the beginning of the pandemic in 2020, NSUHS has operated immediate care facilities that offered clinical evaluation and RT-PCR testing to outpatients, with results usually available within 24 hours. At the time of this study, patients reporting at least one symptom consistent with possible COVID-19 infection were eligible for RT-PCR testing. NSUHS protocols followed Centers for Disease Control (CDC) guidelines to identify symptoms consistent with COVID-19 infection, which included: fever, chills, cough, shortness of breath or difficulty breathing, fatigue, muscle or body aches, headache, new loss of taste or smell, sore throat, congestion or runny nose, nausea, vomiting, or diarrhea [2]. Eligible patients received a nasopharyngeal or oropharyngeal swab collection for COVID-19 RT-PCR testing. Patients for the study were recruited between June 30, 2020 and October 5, 2020.

According to NSUHS protocols at the time of this study, all patients with a positive COVID-19 RT-PCR result received a phone call with the test result from a member of the infection control team or a healthcare provider in the immediate care division. During this call, patients were asked about their willingness to be contacted about COVID-19 research studies for which they might qualify. If they agreed to be contacted, a CITI-trained [3] study team member reviewed the electronic medical record for eligibility. Only patients who reported mild or moderate symptoms were recruited for the current study. The National Institutes of Health (USA) classifies mild illness as including any sign or symptom consistent with COVID-19 but without shortness of breath, dyspnea, or abnormal chest imaging. Moderate illness is defined in patients who report evidence of lower respiratory disease, such as shortness of breath or abnormal chest imaging, but who have a SpO2 of at least 94% [4].

We excluded individuals who presented with severe symptoms or signs such as SpO2 < 94%, or who required emergency department evaluation or hospitalization at any point during their illness course, or who were high risk by age (age of 65 or older), or who were pregnant. Eligible patients were contacted, and the purpose of the study was explained. If interested in participation, informed consent was sent electronically and an initial study visit was scheduled. The informed consent was reviewed at the beginning of the initial study visit to answer questions, ensure understanding and obtain a hard copy signature.

Study participants had nasopharyngeal swabs collected every 3–5 days until the participant had two consecutive negative RT-PCR tests. Samples were transported within four hours of collection, and RT-PCR testing was done using the Abbott m2000 or Abbott Alinity m EUA reagent kits, or an in-house version of the CDC assay [5, 6] on a LC480 II instrument (Roche). The in-house assay was modified from the CDC protocol in that the assay was run on the LC480 II instrument rather than the ABI 7500 Fast Dx (Applied Biosystems). Cycling parameters were not changed. All four recommended targets (N1,N2, N3, RP) were initially validated, but the N2 and N3 targets were dropped as the CDC updated their recommendations. For the duration of this study, only the N1 and RP targets were used. The N1 target provided robust detection of all known circulating strains of SARS-CoV-2 while RP provided an internal control for specimen quality and inhibition. Extensive cross-platform comparisons were performed using direct patient specimens and serial dilutions of available standards, including quantified in vitro transcribed RNA provided by the Illinois Department of Public Health, in vitro transcribed RNA from BEI Resources (NIAID), and a commercially available control containing RNA fragments spanning regions of the ORF1a, RdRp, S, E, and N genes packaged into a viral protein coat (Seracare). These studies indicated the Ct values on the Abbott Alinity and LC480 were nearly identical, while the Abbott m2000 values were consistently offset by 10 cycles. Ct values from the m2000 were therefore adjusted accordingly for data analysis. The LOD for all three assays was 100 copies/mL. This was initially established for the NorthShore in-house assay using the above referenced standards and confirmed during the cross-platform comparisons. This level translates to Ct values of 36–37 for the LC480 and Alinity assays and 26–27 for the m2000 assay.

We used the RT-PCR test results and cycle threshold (Ct) to estimate viral load. Ct is the number of cycles required to pass the threshold for positivity and is a semiquantitative measure that is inversely correlated with viral genomic load. While Ct counts can vary by instrument, a typical RT-PCR assay runs a maximum of 40 cycles to detect the presence of viral genetic material in a sample [7].

A symptom survey (including fever, cough, congestion, sore throat, runny nose, muscle aches, loss/decrease taste or smell, chills/shaking, headache, diarrhea, nausea, decreased appetite) was completed at each visit (performed every 3–5 days) or until the patient had two consecutive negative symptom surveys (Survey is included as S1 File). During weeks 2, 4 and 8, participants had blood collected by venipuncture for serologic testing using the Roche COVID SARS-COV-2 Total IgM/ IgG assay. The last patient completed study data collection on November 30, 2020.

## Statistical methods

We carried out a patient-level analysis by first describing the distribution of their sociodemographic characteristics and the characteristics of their COVID-19 illness and testing statistics. To assess the association of patient’s COVID-19 symptoms and test results with duration of illness, we calculated duration as the difference between the initial diagnosis date and the date of the first of two negative test results. Next, we modeled the association using unadjusted general linear regression models to calculate effect estimates and 95% confidence intervals per cycle count higher and for a 1 symptom higher. Effect estimates were not adjusted given the sample size. Means were compared by t-test and medians were compared by Wilcoxon exact test. All analyses were carried out using SAS version 9.2 (SAS Institute, Cary, NC) between December 30, 2020 and February 15, 2021. Statistical significance was determined at p<0.05.

## Results

### Demographic and clinical characteristics

**Fig 1:**
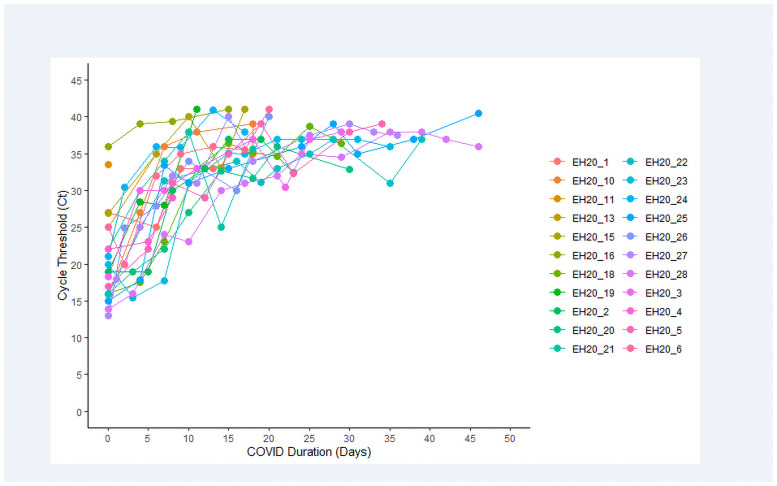
Viral load evolution for each patient. Cycle count, measured in samples collected every 3–5 days from first positive RT-PCR test to first of two negative RT-PCR tests, in patients with complete data.

Table 1 shows age, gender, and clinical characteristics of the 25 patients (nine females) who were enrolled. Median age was 28 years, with range from 19–58. All patients reported at least one symptom, with a median of six symptoms. The most common symptoms were congestion (n = 19, 76%), headache (n = 19, 76%), gastrointestinal upset (n = 17, 68%), muscle aches (n = 17, 68%) and loss of taste or smell (n = 17, 68%). Nine participants (36%) reported shortness of breath, which was the least commonly reported symptom, and these patients were categorized as “moderate” due to respiratory symptoms (more information below). Patients reported symptoms persisting from a minimum of 6 days to a maximum of 67 days, with a median duration of 18 days. Viral load evolution for each patient who had complete follow-up is shown in Fig 1.

**Table 1:** Clinical characteristics of 25 patients with mild or moderate COVID-19.

Age (Median, range, years)	28 (19–58)
Gender (% Male)	16 (64%)
Symptoms (Count, % Present)	
Subjective fever Yes/No	12 (48%)
Cough	13 (52%)
Congestion	19 (76%)
Sore throat	13 (52%)
Rhinorrhea	13 (52%)
Shortness of breath	9 (36%)
Muscle Aches	17 (68%)
Loss or decrease of taste or smell	17 (68%)
Chills or shaking	14 (56%)
Headache	19 (76%)
Nausea, anorexia or other gastrointestinal symptoms	17 (68%)
Duration of symptoms (any), Median	18.0
Number of symptoms, Median	6.0
Days from onset of symptoms to first PCR test, Median	2.0
Cycle threshold (Ct) at first PCR test, Median	20.0
Days from symptom onset to first of 2 negative PCT, Median	24.0
Antibody test positive at 2 weeks after symptom onset	15/25 (60%)
Antibody Test positive at 4 weeks after symptom onset	23/23(100%)

### Association of symptoms with RT-PCR results (Ct)

**Fig 2:**
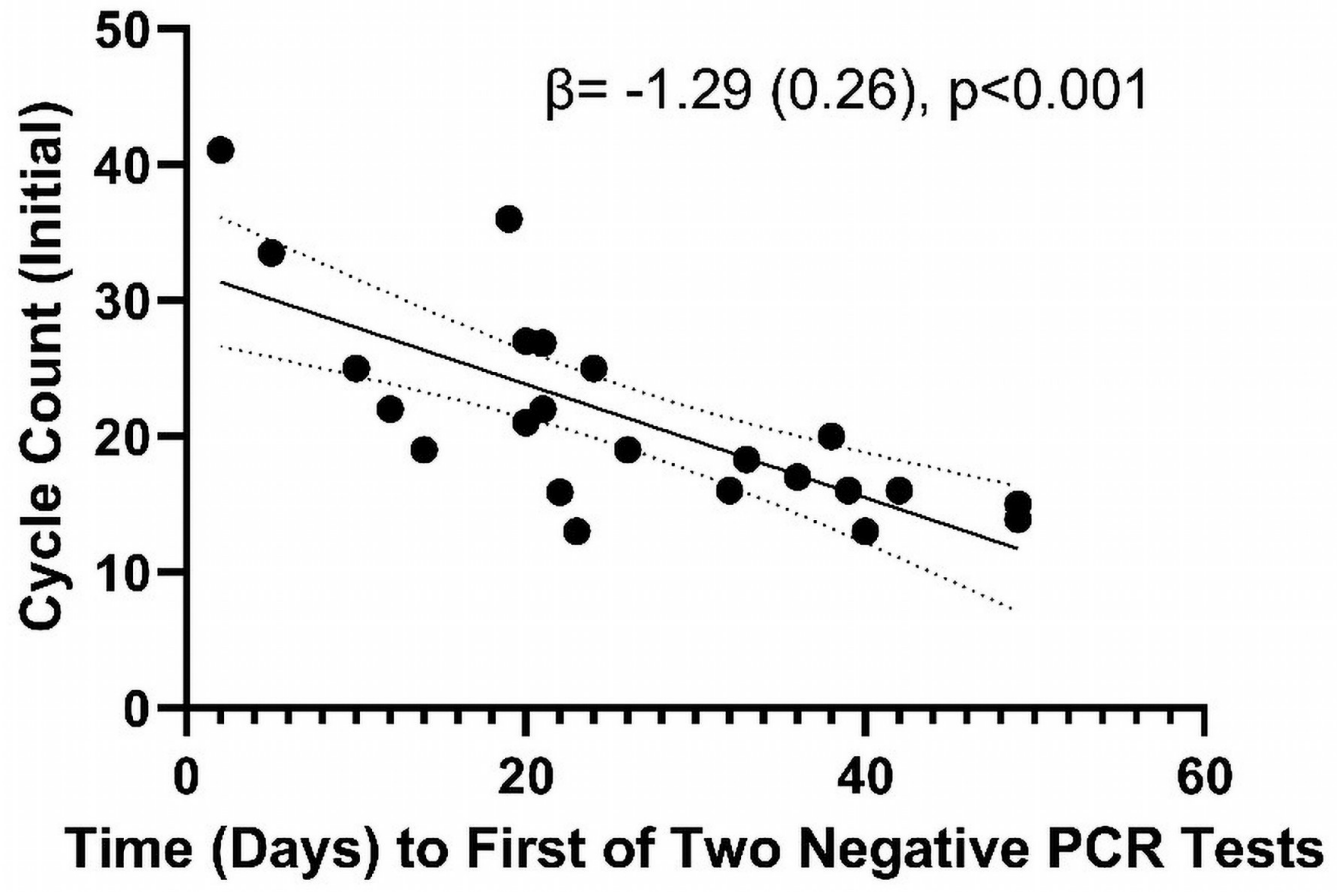
Duration of positive RT-PCR test for SARS-CoV-2 according to initial cycle threshold (Ct). Duration of positive RT-PCR test was negatively correlated with initial cycle threshold (Ct). Longest duration of positive test in this study was 49 days from symptom onset.

**Fig 3:**
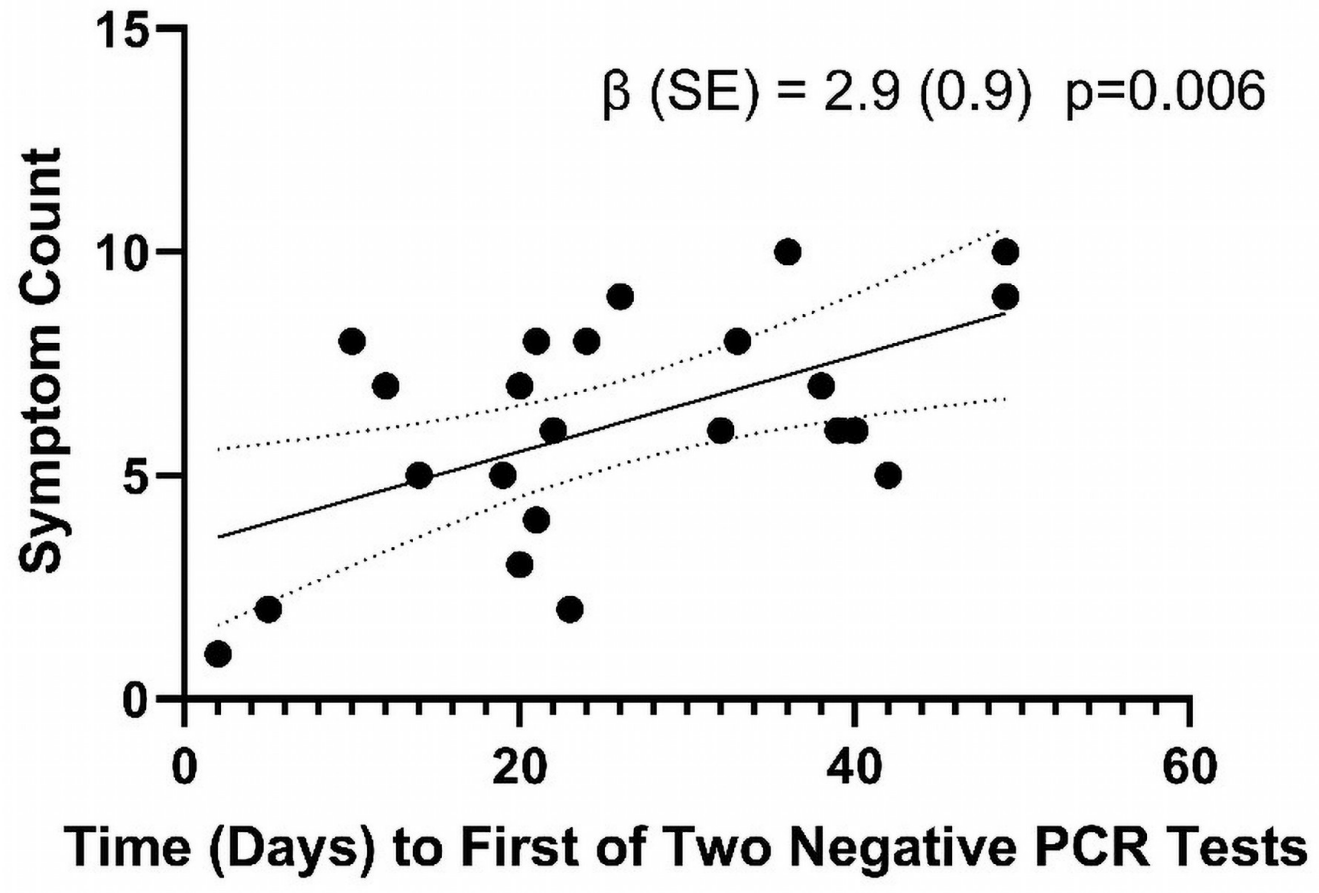
Duration of positive RT-PCR test for SARS-CoV-2 according to number of symptoms. Duration of positive RT-PCR test was positively associated with number of symptoms. Patients with more symptoms had a positive RT-PCR test significantly longer than patients with fewer symptoms.

Following exclusion of 2 patients due to early dropout, we determined the association of initial Ct count with duration of illness. The longest duration of positive RT-PCR test was 49 days from first positive PCR test (Median = 24, minimum 5 days). Initial Ct was significantly associated with longer symptom duration (β = -1.3, SE = 0.3, p<0.01 per 1 cycle higher) of RT-PCR positivity (Fig 2). We also determined the association of number of symptoms with duration of RT-PCR positivity. For each additional symptom, duration of PCR positivity was longer (β = 2.8, SE = 0.9, p = 0.01 per 1 symptom greater) (Fig 3).

### Comparison of patients with moderate disease versus mild disease

Nine patients reported dyspnea and therefore were considered to have “moderate” COVID-19 compared to 15 patients with complete data for follow-up and no dyspnea. Comparing patients without dyspnea to those with, there were no significant differences in first Ct value (medians, 19 and 22, respectively, p = 0.24), in duration of positive PCR test (medians, 24.5 days and 22.5 days, p = 0.47), and in duration of symptoms (medians, 24.0 and 24.0, p = 0.70). Additional results for means of these measurements are shown in S1 Table.

### Serology data

All patients were asked to provide blood samples for serologic antibody testing during study visits in weeks 2, 4 and 8. During week 2, samples were collected a median of 13 days after the onset of symptoms (range 10–20 days). Of the samples collected during week 2 in all 25 patients, 15 (60%) were positive for SARS-CoV-2 antibodies.

During week 4, blood samples were collected a median of 28 days after symptom onset (range 24–34 days). Of the 23 patient samples collected during study week 4, all 23 were positive for SARS-CoV-2 antibodies.

During week 8, samples were collected a median of 54 days after the onset of symptoms (range 52–65 days) in 21 patients, and all 21 samples that were collected were positive for antibodies.

All available data for all patients, including symptoms, Ct, and antibody results are contained in S2 Table.

## Discussion

This study of symptomatic mild or moderate COVID-19 outpatients resulted in several findings. First, we found a correlation between initial viral load, as measured by Ct values, and the duration of positive RT-PCR test results. Second, we found that the number of symptoms that patients experienced was also correlated with duration of positive RT-PCR tests. Third, there was considerable variability in the Ct values of outpatients presenting to an immediate care clinic. This number (Ct value) is rarely reported to patients or physicians. To our knowledge, this is the first study of patients with mild-to-moderate COVID-19 in which serial respiratory samples were collected until RT-PCR tests became negative. In these non-hospitalized patients, we found that positive RT-PCR tests persisted for as long as 49 days after initial positive test.

There is a small number of previous reports in symptomatic patients with mild or moderate COVID-19. Zou, et al reported on 17 mildly symptomatic patients from China [8]. Similar to our study, Zou, et al tested patients serially for RT-PCR. They reported that all patients became RT-PCR negative by day 18 after onset of symptoms. This finding is inconsistent with our study and also inconsistent with findings from other studies, described below. Wajnberg, et al. [9] performed a repeat (total of 2) RT-PCR test in 182 mildly symptomatic outpatients from New York. The time interval between first and second tests was a median of 10 days (7–12, IQR). They reported that 62% of the patients were negative for RT-PCR at the second test. They did not follow patients until all were negative. However, based on a larger number of samples collected once, they found that PCR positivity was detected for up to 28 days following symptom resolution. This set of findings is largely consistent with ours though they did not perform serial tests until RT-PCR negativity in all patients. In Wajnberg, et al, of 624 patients with confirmed SARS CoV-2 infection who had serologies done after 4 weeks, all but 3 seroconverted. In our study, by 4 weeks, 100% of the 21 patients we followed serially had seroconverted.

Yilmaz, et al. [10] did serial respiratory sample collections in 39 patients with mild disease from Sweden. Median duration of viral shedding as measured by RT-PCR was 24.0 days. Similar to our study, they found a moderate correlation between number of days with symptoms and number of days with viral RNA shedding (Pearson r = 0.34, p = 0.05). Most patients (26/34) continued to have positive RT-PCR tests after resolution of symptoms, similar to our finding. Other studies in mildly to moderately sick patients have not included serial RT-PCR tests [9, 11–14].

This study has both strengths and limitations. One of the strengths is the focus on mildly or moderately ill patients, in contrast to the numerous reports in hospitalized patients. We found only 8 previous studies focused on symptomatic outpatients despite the prevalence of this form of COVID-19. Another strength of this study is the serial testing for RT-PCR and careful tracking of symptoms in all patients. The major weakness of the study is its small numbers, but our findings, taken along with those in other reports, forms a picture of viral load dynamics in mildly or moderately ill patients.

## Conclusions

Our findings indicate that viral load tests by Ct value can be used to estimate duration of “viral shedding” by RT-PCR tests and resolution of symptoms. Since positive RT-PCR tests can be found for up to 49 days, RT-PCR test results should not be used as a guide to return to work or other daily activities.

## Supporting information

S1 File Symptom survey. (DOCX)

S1 Table Table of results for patients with and without dyspnea. (DOCX)

S2 Table Detailed results for all patients, with symptoms, Ct, viral culture and antibody results. (XLSX)
