# Supplementary material for: Clinical characteristics and viral load dynamics of COVID-19 in a mildly or moderately symptomatic outpatient sample
Source: PLoS One. 2021 Oct 21;16(10):e0258970. doi: 10.1371/journal.pone.0258970 (PMC8530348; doi:10.1371/journal.pone.0258970)
Supplement: S1 File — (DOCX) [file pone.0258970.s001.docx]

**Supplement S1. Symptom Survey**


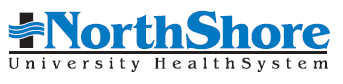
 Survey # ______

COVID-19 Data Collection Symptoms Survey

**Date:**__________  **Name:**______________________ **DOB**:_________

Please respond: Yes/No/Never to each question.

- If your answer is “YES” and this is a change from your last survey, please provide the date of when this symptom started.
- If your answer is “NO” and this is a change from your last survey, please provide the date of the last time you experienced this symptom.

Fever: Yes No Never Date: ________

Cough: Yes No Never Date: _________

Congestion: Yes No Never Date: ________

Sore throat: Yes No Never Date: ________

Runny nose: Yes No Never Date: _______

Shortness of breath: Yes No Never Date: ________

Muscle aches w/ flu-like symptoms: Yes No Never Date:_______

Loss/decrease taste or smell: Yes No Never Date: ________

Chills/shaking: Yes No Never Date: ________

Headache: Yes No Never Date: ________

Diarrhea, nausea, decreased appetite, or any GI symptoms:

Yes No Never Date: ________
