# Supplementary material for: Clinical characteristics and viral load dynamics of COVID-19 in a mildly or moderately symptomatic outpatient sample
Source: PLoS One. 2021 Oct 21;16(10):e0258970. doi: 10.1371/journal.pone.0258970 (PMC8530348; doi:10.1371/journal.pone.0258970)
Supplement: S1 Table — (DOCX) [file pone.0258970.s002.docx]

| S1 Table. Table of results for patients with and without dyspnea (shortness of breath) | | | | |
| --- | --- | --- | --- | --- |
| Patient No. | Shortness of Breath (N/Y) | First Ct | Duration of Positive PCR | Duration of symptoms |
| 1 | N | 27 | 21 | 12 |
| 2 | N | 19 | 28 | 14 |
| 4 | N | 22 | 14 | 14 |
| 10 | N | 16 | 24 | 21 |
| 15 | N | 27 | 27 | 19 |
| 17 | N | 27 | N/A | 21 |
| 18 | N | 16 | 33 | 28 |
| 19 | N | 19 | 15 | 36 |
| 20 | N | 16 | 39 | 6 |
| 22 | N | 16 | 46 | 16 |
| 23 | N | 20 | 39 | 18 |
| 24 | N | 21 | 21 | 9 |
| 26 | N | 13 | 24 | 18 |
| 27 | N | 13 | 41 | 19 |
| 28 | N | 14 | 52 | 44 |
| Mean |  | 19.1 | 30.3 | 19.7 |

| Patient No. | Shortness of Breath | First Ct | Duration of Positive PCR | Duration of symptoms |
| --- | --- | --- | --- | --- |
| 3 | Y | 18 | 36 | 23 |
| 5 | Y | 17 | 40 | 13 |
| 6 | Y | 25 | 28 | 19 |
| 11 | Y | 34 | 7 | 13 |
| 13 | Y | 25 | 15 | 23 |
| 14 | Y | 21 | 24 | 9 |
| 16 | Y | 22 | 27 | 7 |
| 21 | Y | 22 | 23 | 25 |
| 25 | Y | 15 | 50 | 67 |
| Mean |  | 22.1 | 27.8 | 22.1 |
| p-value |  | 0.18 | 0.63 | 0.34 |
